# Entorhinal cortex and parahippocampus volume reductions impact olfactory decline in aged subjects

**DOI:** 10.1002/brb3.2115

**Published:** 2021-03-26

**Authors:** Natsuko Iizuka, Yuri Masaoka, Satomi Kubota, Haruko Sugiyama, Masaki Yoshida, Akira Yoshikawa, Nobuyoshi Koiwa, Motoyasu Honma, Keiko Watanabe, Shotaro Kamijo, Sawa Kamimura, Masahiro Ida, Kenjiro Ono, Masahiko Izumizaki

**Affiliations:** ^1^ Department of Physiology Showa University School of Medicine Tokyo Japan; ^2^ Deparment of Neurology Showa University School of Medicine Tokyo Japan; ^3^ Sensory Science Research Kao Corporation Tokyo Japan; ^4^ Department of Ophthalmology Jikei Medical University Tokyo Japan; ^5^ Department of Health and Science University of Human Arts and Sciences Saitamaken Japan; ^6^ National Hospital Organization Mito Medical Center Ibaragiken Japan

**Keywords:** brain volume, entorhinal cortex, memory, olfactory decline, parahippocampus

## Abstract

**Introduction:**

Pathological abnormalities first appear in the medial temporal regions including entorhinal cortex and parahippocampus in patients with Alzheimer's disease. Previous studies showed that olfactory decline in elderly subjects was associated with volume reductions in the left hippocampus and left parahippocampus without cognitive impairment. The aim of this study is to investigate the link between olfaction and volume reductions in the medial temporal regions including the parahippocampus, entorhinal cortex, and hippocampal subfields.

**Method:**

27 elderly subjects and 27 young controls were measured olfaction acuity, cognitive function, and structural magnetic resonance imaging. Image processing and gray matter volumetric segmentation were performed with FreeSurfer. Volume data were analyzed with SPSS Statistics software.

**Results:**

Interesting results of this study were that volume reduction in the entorhinal cortex was not directly linked with declining olfactory ability. Volume reduction in the left entorhinal cortex was correlated with volume reduction in the left parahippocampus and dentate gyrus. However, left parahippocampus volume reduction had the greatest impact on olfactory decline, and the entorhinal cortex and dentate gyrus might additionally contribute to olfactory decline.

**Conclusion:**

Our results indicate that olfactory decline may be directly reflected in the medial temporal regions as reduced parahippocampus volumes, rather than as morphological changes in the entorhinal cortex and hippocampus. The parahippocampus may play an important role in the association between memory retrieval and olfactory identification.

## Graphical abstract

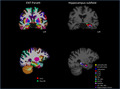

## 1 INTRODUCTION

Olfactory impairment is the first sign of Alzheimer's disease (AD) and Parkinson's disease (Doty et al., 1988; Hawks, 2003). Neuroimaging studies have revealed that pathological changes in these patients begin in the amygdala (AMG) and hippocampus (HI) and especially the parahippocampus (para‐HI) and entorhinal cortex (ENT), which having an important role in olfactory recognition and identification (Doty et al., 1988; Hawks, 2003; Mesholam et al., 1998). In addition to neurodegenerative symptoms, patients with mild cognitive impairment (MCI) have also olfactory (Roberts et al., 2016) as well as healthy elderly subjects (Kubota et al., 2020).

The ability to identify a specific odor involves two capacities. First, the chemical signature must be transmitted from the olfactory bulb to the ENT, which has direct projection to the HI (Lavenex & Amaral, 2000). The second is olfactory identification ability, that is, the ability to categorize the odor by a single name and recall the memory of the odor through engaging the neural network connecting the HI and para‐HI to the orbitofrontal cortex (OFC), which is a center for odor recognition (Rolls, 2001). Olfactory impairment could be caused by abnormalities in the “input” or “output” of this system or both factors, depending on the functional state and connectivity in the brain.

A previous study reported that the detection threshold for odor was intact in patients with AD, but recognition of the odor was specifically impaired (Masaoka et al., 2013). Accordingly, we hypothesized that the “output’ portion of olfactory ability might be impaired and the “input’ ability remained intact. Based on this hypothesis, we used structural neuroimaging data to investigate whether volume changes in olfactory areas, AMG, HI, OFC, para‐HI, rectus, and middle frontal cortex (MFC) were associated with olfactory decline in aged subjects with normal cognitive function (Kubota et al., 2020). Although normal elderly subjects had reduced bilateral AMG, HI, OFC, and MFC and right rectus volumes compared with younger subjects, these reductions were not correlated with olfactory ability. In contrast, no difference was found in the para‐HI volume between the two age groups. However, a significant correlation was observed between para‐HI volume and olfactory ability in aged subjects, indicating that para‐HI volume changes exhibit individual differences in elderly subjects. Therefore, we suggest that declining olfactory ability begins with para‐HI volume reduction (Kubota et al., 2020).

The para‐HI may be the key area for olfactory identification; however, the ENT is anatomically near the para‐HI and has been reported to exhibit initial pathological changes in patients with dementia (Khan et al., 2014; Koychev et al., 2017;). Indeed, the ENT has a direct projection to the HI including the dentate gyrus (DG), CA3 and CA1 regions and subiculum (Amaral & Whitter, 1989). In turn, the CA1 region and subiculum project back to the ENT (Kobayashi & Amaral., 2003; Lavanex & Amaral, 2000; Rosene & Van Hoesen, 1977), playing the role of the hub of the hippocampal formation. Regarding the olfaction site, the ENT is one of the primary olfactory areas that directly projected from the olfactory bulb (Sobel et al., 2000). Accordingly, pathological abnormalities in the ENT will impair transmission of information from the olfactory bulb (olfactory input) and the relay of signals to the HI for memory formation as well as projection back to the neocortex (output abilities). However, it remains unclear whether olfactory impairment is caused by disruption of the relationship between the ENT and hippocampal subfields or association between the ENT and para‐HI in the beginning of olfactory decline before the onset of dementia.

A previous study (Kubota et al., 2020) reported that the para‐HI volume, which included the ENT volume, predicted olfactory recognition ability. In this study, we separately measured the para‐HI and ENT volumes, as well as that of the hippocampal subfields, and investigated a relationship between volume changes in these structures and olfactory ability in elderly subjects. To explore this idea, we hypothesized a link between olfactory identification and the olfactory circuit based on our path analysis results.

## 2 METHODS

### 2.1 Participants

The subjects included in this study comprised a subset of subjects from a previously published study (Kubota et al., 2020) who had structural magnetic resonance imaging (MRI) data, olfactory, and cognitive test scores available. Demographic information is shown in Table 1. Elderly subjects (*N* = 30) who had no mild cognitive impairments participated in the study. All elderly subjects were living independently. Young healthy controls (*N* = 27) including students and office staff members were participated. The exclusion criteria for all participants were (1) a history of head injury or seizures; and (2) diagnosis of a neurological disorder. Three elderly subjects were excluded because of previous cerebral infarction (2 subjects) or subarachnoid hemorrhage (1 subject), resulting in a final total of 27 elderly subjects. Total of 27 elderly subjects were participated in this study.

**TABLE 1:** Demographic data of young healthy controls and elderly subjects

	Young	Elderly
No. Subjects (gender)	27 (Female, 10/Male, 17)	27 (Female, 14/Male, 13)
AGE	37.8 ± 9.1	73.7 ± 5.4***
Handedness(R/L)	(Right,26, Left1)	(Right, 26, Left, 1)
Year of education	17.8 ± 2.9	13.8 ± 2.5***
MoCA	28.6 ± 2	25.5 ± 2.2***
Olfactory Detection Level	−0.4 ± 0.7	0.9 ± 0.7***
Olfactory Recognition Level	0.2 ± 0.7	2.3 ± 0.6***

Note

Data modified from Kubota et al. (2020).

*** *p* < .0001, **p < .001, *p < .01.

This study was approved by the institutional review board of the Showa University Hospital and the Ethical Committees of Showa University School of Medicine, and all participants provided informed consent prior to participation. All experiments were conducted in accordance with the Declaration of Helsinki (https://www.wma.net/policies‐post/wma‐declaration‐of‐helsinki‐ethical‐principles‐for‐medical‐research‐involving‐human‐subjects/).

### 2.2 Olfactory and cognitive assessments

T&T olfaction test was used to test all subjects’ odor detection acuity and odor recognition acuity (Takasuna Co., Ltd.). The T&T test is a Japanese standardized olfactory ability test and often used in otorhinolaryngology for diagnosing patients with anosmia and hyposmia in Japan. The T&T test has been used for patients with neurodegenerative disorder including Parkinson's and Alzheimers’ disease (Masaoka et al., 2013). The test had correlation with the University of Pennsylvania Smell Identification Test (Kondo et al., 1998). The test has five odors. Odor A is the odor of rose (*β*‐phenyl ethyl alcohol); odor B is the odor of caramel (methyl cyclopentenolone); odor C is the odor of rotten food (iso‐valeric acid); odor D is the odor of peach (*γ*‐undecalactone); odor E is the odor of lichen refuse (skatole). Each odor was dissolved in propylene glycol with eight different concentrations. Subjects were tested beginning with the lowest concentration and were repeated with higher concentrations. The odors were presented randomly but at the same concentration in each trial. The odor was perceived but not identified was considered the “odor detection.” As increasing odor concentration, the subject could identify the odor. The subjects were required to naming each odor and describe the type of odor. Odor was first identified considered as the “odor recognition.” Final scores were expressed as the average of all threshold concentration level and the average of all recognition concentration level (odor recognition). Higher scores mean lower olfactory detection and recognition abilities. Cognitive function was measured using the Japanese version of the Montreal Cognitive Assessment (MoCA‐J) (Nasreddine et al., 2005).

### 2.3 MRI data acquisition

All MRI data were measured at Ebara Hospital using a Siemens 3 Tesla MAGNETOM Prisma fit (Siemens) with a 64‐channel phased‐array coil. The anatomical scan was measured following parameters: T1‐weighted 3D MPRAGE sequence (9 degree flip angle; TR 2300 ms; TE 2.98 ms; matrix size 256 × 256; field of view 256 mm; 176 slices with a voxel size of 1 mm^3^.)

### 2.4 MRI data analysis

Images were processed using FreeSurfer (Version 6.0) automated neuroanatomical segmentation software (http://surfer.nmr.mgh.harvard.edu). Gray matter volumes were determined on each T1‐weighted scan with the recon‐all script on FreeSurfer. FreeSurfer automatically performs volumetric segmentation (Fischl et al., 2002), cortical surface reconstruction (Dale et al., 1999; Fischl & Dale, 2000; Fischl et al., 1999), and parcellation (Desikan et al., 2006; Fischl et al., 2004) after preprocessing for images including motion correction, removing nonbrain tissue, normalization with nonuniform intensity, affine registration to Montreal Neurological Institute (MNI) space, and Talairach transformation. Visual inspections for all boundaries were confirmed with a graphic tools of Freeview and TkMedit installed FreeSurfer after affine registration to MNI space. Two trained neurologists inspected all volumetric segmentations for accuracy and edited minor changes with the TkMedit editing tool and he images were re‐run with the recon‐all script. We performed manual editing within the range of removing nonbrain tissue included within the cortical boundary.

**FIGURE 1:**
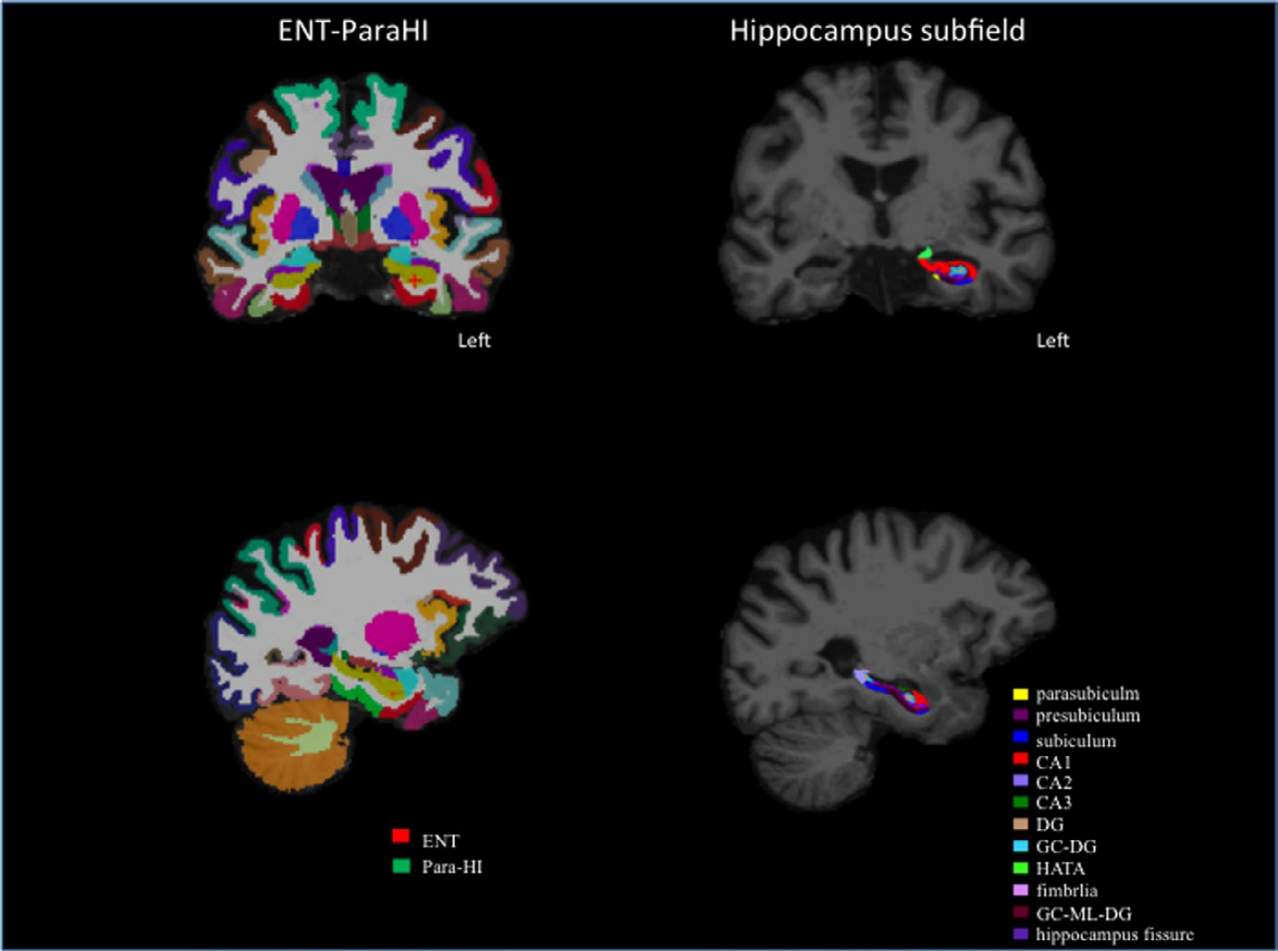
Left: Visualization of brain segmentation in a single subject with FreeSurfer. The entorhinal cortex (ENT) is indicated in red, and the parahippocampus (para‐HI) is indicated in green. Right: Visualization of hippocampal subfield segmentation in a single subject with each subfield represented by a different color. DG, dentate gyrus, GC‐DG, granule cell layer of the dentate gyrus, HATA, HI‐AMG transition area, GC‐ML‐DG, granule cells in the molecular layer of the dentate gyrus

After successful reconstruction of the whole hippocampus and surrounding subcortical regions, we used revised version of the automated subregion parcellation protocol (FreeSurfer Version 6.0; Iglesias et al., 2015) which based on a prior version (FreeSurfer Version 5.3; Van Leemput et al., 2009). The prior version can predict the locations of eight hippocampus subregions including the CA1, CA2/3, DG, subiculum, presubiculum, fimbria, hippocampus tail, and hippocampus fissure by combining a single probabilistic atlas with high resolution and T1‐weighted in vivo manual segmentations. The new version (Iglesisas et al., 2015) predicts the location of twelve hippocampus subregion including the parasubiculum, granule cell layer of the dentate gyrus (GC‐DG), granule cells in the molecular layer of the DG (GC‐ML‐DG), HI‐AMG transition area (HATA) as well as prior eight region (Figure 1). The new version predicts twelve hippocampus location with refined probabilistic atlas built based on combined the manual delineations of hippocampus formation using ultra‐high resolution, ex *vivo* MRI scans, and manual annotation of the substructure including the amygdala and cortex from T1‐weighted with 1mm resolution MRI scans (Iglesisas et al., 2015).

In addition to volume measurements of the medial temporal regions, olfactory sulcus volume was also measured because it has been reported to reflect olfactory bulb volume (Delon‐Martin et al., 2013).

For analyses of the brain volume, intracranial volume (ICV) was used as a covariate. In the previous report (Kubota et al., 2020), we compared the results of volumetric group comparison between analysis using ICV measured with FreeSurfer and ICV measured with the Statistical Parametric Mapping 12 (SPM12) program (Ashburner & Friston, 2005). The results indicated no significant difference in the ICVs calculated by the two methods. In this study, we used the ICV calculated by FreeSurfer as a covariate for the statistical analysis.

### 2.5 Data analysis

Group differences in left and right hippocampal subfields were assessed using analysis of covariance (ANCOVA) with sex, years of education, and ICV included as covariates. Group comparisons of olfactory sulcus volumes were also performed using ANCOVA, with sex, years of education, and ICV as covariates. Volume data analyzed with FreeSurfer were entered into SPSS Statistics software (IBM SPSS Statistics, version 23.0, IBM Corp).

Before performing path analysis, partial correlation and multiple regression analyses were performed to refine our hypothesis. Partial correlation tests within‐group were conducted between olfactory recognition scores and the para‐HI, ENT, and hippocampal subfields, covarying with differences in age, years of education, and sex. Then, multiple regression analysis was performed using the para‐HI as an independent variable and the ENT and hippocampal subfields as dependent variables. Next, path analysis was conducted to examine the impacts of the para‐HI, ENT, and hippocampal subfields on olfactory recognition and the MoCA‐J scores in both groups. The structural equation modeling program employed (AMOS version 23; IBM SPSS Statistics) specified the statistical significance of path coefficients. Path analyses can provide estimates of the relative importance and significance of hypothesized causal connections between sets of variables. For example, the effect of the association between the ENT and DG or between the para‐HI and DG on olfactory recognition could be investigated. Path analysis can also estimate the significant values of the indirect path (for example, the effect of the ENT volume on olfactory ability mediated through the para‐HI).

At first step, we added path between all volumes of all ROIs, the MoCA‐J and olfactory recognition scores. Then nonsignificant path was eliminated to create the final path model. We conducted assessments to confirm the validity of the models with observation of goodness of fit index (GFI) and Bollen–Stine bootstrap evaluations. A GFI close to 1 and a Bollen–Stine bootstrap *p* value > .05 were adopted for the model.

For exploratory analyses, partial correlations between olfactory sulcus volumes and olfactory detection or recognition scores were analyzed within groups, with age, years of education, and sex as covariates.

## 3 RESULTS

Table 1 shows the demographic data. There were significant differences in age, years of education, and MoCA‐J between HCs and elderly subjects (the detailed statistical results were reported in Kubota et al., 2020). Lower olfactory detection ability was observed in elderly subjects than young HCs. Impaired olfactory recognition was observed in five elderly subjects, and other 22 subjects showed lower olfactory recognition abilities than young HCs (Kubota et al., 2020). All elderly subjects had hyposmia, which is a decreased sense of smell. In the current study, two types of hyposmia were observed. Five elderly subjects who impaired olfactory recognition were able to detect odors, but had difficulty identifying each odor, even when they were tested with higher odor concentrations. The other 22 subjects were able to both detect and identify odors, but their detection and recognition abilities were significantly worse than those of young HCs. That is, these 22 elderly subjects were only able to detect and recognize odors at higher odor concentrations compared with the young HCs. But all subjects had no complain about declining or loss of olfactory ability in daily life.

Comparisons of volumes of olfactory brain regions AMG, HI, OFC, rectus, and MFC volumes were reported in the previous study (Kubota et al., 2020). Elderly subjects had smaller intracranial, whole brain, right rectus, bilateral AMG, HI, OFC, and MFC volumes than younger subjects (the detailed statistical results were reported in Kubota et al., 2020). Table 2 shows statistical differences for each brain region between elderly and young HCs. The left para‐HI, left ENT, and right ENT had no differences between the elderly subjects and young HCs. There were statistically lower volumes in most hippocampal subfields in elderly subjects compared with younger HCs, except for the right CA3 area, right DG, left subiculum, and left parasubiculum. All statistical results are shown in Table 2.

**TABLE 2:** Comparison of brain regions between healthy controls and elderly subjects

Brain regions	Young	Elderly	Statistic		
			*F* value	*p* value	Partial eta squared
L‐para‐HI	2,151 ± 323	1886 ± 290	1.2	.27	0.02
R‐para‐HI	2,207 ± 246	1863 ± 285	4.5	.04*	0.08
L‐ENT	1801 ± 374	1666 ± 242	0.04	.82	0.001
R‐ENT	1851 ± 290	1718 ± 298	0.28	.59	0.006
L‐CA1	634 ± 47	547 ± 59	10.23	.002*	0.17
R‐CA1	671 ± 69	592 ± 83	3.94	.05*	0.07
L‐CA3	211 ± 26	178 ± 24	5.36	.02*	0.09
R‐CA3	219 ± 31	202 ± 33	0.001	.99	0.001
L‐DG	262 ± 25	220 ± 28	10.31	.002*	0.17
R‐DG	261 ± 27	237 ± 34	0.64	.42	0.01
L‐GC‐DG	307 ± 28	255 ± 34	12.33	.001*	0.2
R‐GC‐DG	307 ± 31	271 ± 40	2.22	.14	0.04
L‐subiculum	430 ± 92	393 ± 46	0.57	.45	0.01
R‐subiculum	453 ± 34	401 ± 47	10.2	.002*	0.17
L‐presubiculum	308 ± 32	268 ± 35	5.79	.02*	0.11
R‐presubiculum	305 ± 24	254 ± 40	12.98	.001*	0.21
L‐parasubiculum	62 ± 11	51 ± 10	3.28	.07	0.06
R‐parasubiculum	60 ± 13	46 ± 9	8.88	.004*	0.15
L‐HATA	59 ± 8	50 ± 7.7	7.14	.01*	0.12
R‐HATA	60 ± 8	49 ± 9	9.84	.003*	0.16
L‐fimbria	103 ± 22	72 ± 20	16.87	.0001*	0.25
R‐fimbria	91 ± 22	58 ± 23	12.66	.001*	0.2
L‐hippocampus fissure	149 ± 18	159 ± 25	3.73	.05*	0.07
R‐hippocampus fissure	157 ± 28	175 ± 25	7.6	.008*	0.13
L‐hippocampus tail	532 ± 65	451 ± 76	7.19	.01*	0.12
R‐hippocampus tail	581 ± 64	475 ± 82	12.4	.001*	0.2
L‐GC‐ML‐DG	579 ± 38	485 ± 54	19.55	.0001*	0.28
R‐GC‐ML‐DG	596 ± 46	514 ± 68	8.6	.005*	0.14

Abbreviations: DG, dentate gyrus; Entorhinal cortex; GC‐DG, granule cell layer of the dentate gyrus; GC‐ML‐DG, granule cells in the molecular layer of the dentate gyrus; HATA, HI‐AMG transition area; L, left; Para‐HI, parahippocampus, ENT; R, right.

* *p* < .05, **p < .01, *** *p* < .001.

In addition to the aforementioned areas, olfactory sulcus volume was also compared between the two groups. There were no differences in left and right olfactory sulcus volumes between the two groups (left olfactory sulcus volume: young HCs, 954 ± 106, elderly subjects, 826 ± 112, *F* = 3.2, *p* = .08, partial eta squared = 0.06; right olfactory sulcus volume: young HCs, 1,040 ± 146, elderly subjects, 936 ± 106, *F* = 0.53, *p* = .46, partial eta squared = 0.01). For the exploratory analysis, a partial correlation analysis was performed between olfactory sulcus volumes and olfactory recognition/detection scores within the groups. In both groups, there were no correlations between left olfactory sulcus volumes and olfactory detection scores (young HCs, *r *= −.26, *p* = .2, elderly subjects, *r *= −.01, *p* = .96), or between left olfactory sulcus volumes and olfactory recognition scores (young HCs, *r* = .25, *p* = .22, elderly subjects, *r* = .16, *p* = .5). There were also no correlations between right olfactory sulcus volumes and olfactory detection scores (young HCs, *r *= −.13, *p* = .5, elderly subjects, *r *= −.05, *p* = .83), or between right olfactory sulcus volumes and olfactory recognition scores (HCs, *r* = .21, *p* = .32, elderly subjects, *r* = .25, *p* = .28).

### 3.1 Partial correlation and multiple regression analyses were performed to refine our hypothesis

Partial correlations between olfactory recognition scores and para‐HI, ENT, and hippocampal subfield volumes covaried with differences in age, sex, and years of education in elderly subjects (*N* = 22, impaired olfaction subjects were not included). All statistical results are included in Table S1. In brief, the left para‐HI was negatively correlated with olfactory recognition. Olfactory recognition scores were negatively correlated with the right CA1 region, left DG, right DG, left GC‐ML‐DG, right GC‐ML‐DG, left subiculum, right subiculum, left hippocampal tail, left molecular layer of the HI, and right molecular layer of the HI (statistical details in Table S1). Multiple regression analysis was conducted with olfactory recognition as a dependent variable and all areas as independent variables. Stepwise multiple regression analysis revealed that left para‐HI volume was significantly associated with the olfactory recognition score in elderly subjects (*B* = −0.59, *p* = .003). To explore this association, multiple regression analysis was performed with the left para‐HI as the dependent variable and the left hemisphere regions as independent variables. The stepwise multiple regression analysis revealed that the left ENT was significantly associated with the left para‐HI in elderly subjects (*B* = 0.72, *p* = .0001).

### 3.2 Path analysis

**FIGURE 2:**
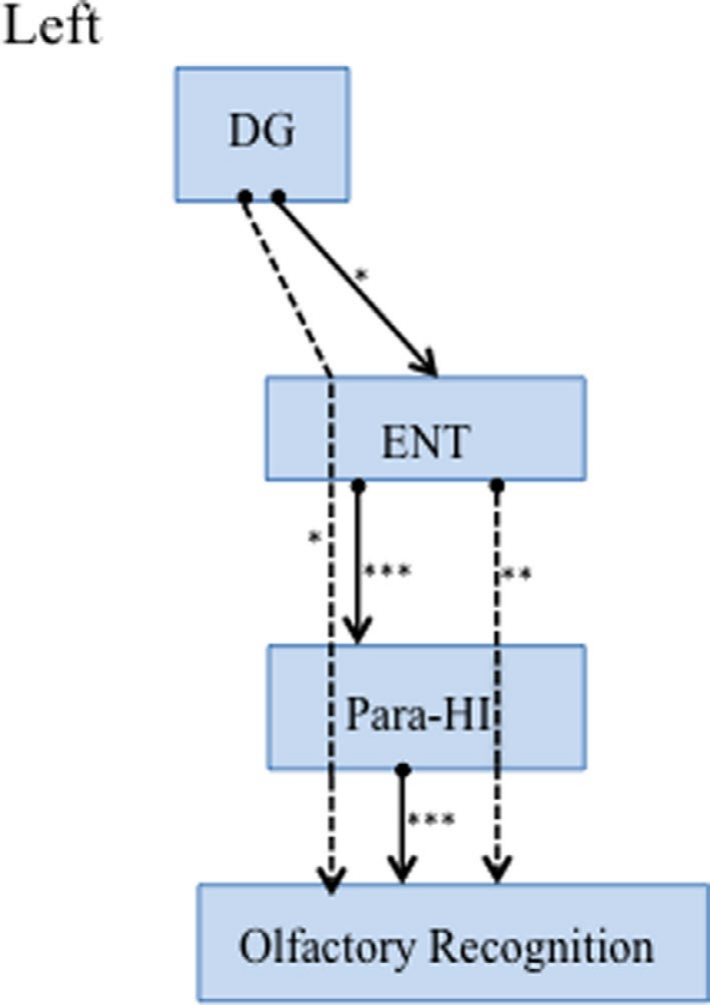
Path diagram and standardized path coefficients of elderly subjects. The solid lines indicate significant standardized direct effects. The dotted lines indicate significant standardized indirect effects. *p < .05, **p < .01, ***p < .0001 Statistical details were indicated in Table 3. DG, dentate gyrus, ENT, entorhinal cortex, para‐HI, parahippocampus

Finally, path analyses were performed to test the predictive ability of interactions of each brain region’ volume for olfactory impairment in elderly subjects (Figure 2). The model fitness was confirmed by the following indices: Bollen–Stine bootstrap, *p* = .07, GFI, 0.9. Decreased olfactory recognition ability was predicted by a small left para‐HI volume (Figure 2). The ENT volume indirectly impacted olfactory recognition via the para‐HI. This indirect path had a greater effect on olfactory recognition than the direct path from the para‐HI. The volume change of the DG predicted ENT volume, and olfactory recognition was affected via ENT and para‐HI volume changes. All statistical details were shown in Table 3. Before finalizing the path model, nonsignificant paths were eliminated. A model that was created before the final path included the important nodes (CA1, CA3, GC‐DC, and subiculum) where a direct link to the DC and ENT was indicated (see Figure S1 and Table S2, which includes the statistical results). Young HCs were tested with the same steps and showed no significant paths (all path, *p* > .05).

**TABLE 3:** Statistical results of path analysis

Standardized direct effect	Standardized regression weight	*p*	
L‐DG ‐ L ENT	0.44	.03*	
L ENT ‐ L‐Para‐HI	0.63	.0001*	
L‐Para‐HI ‐ Olfactory recognition	−0.59	.0001*	

Standardized indirect effect	Lower bunds	Upper bounds	*p*
L‐DG ‐ L para‐HI	−0.006	0.61	.06
L‐DG‐ Olfactory recognition	−0.4	0.002	.05*
L ENT ‐ Olfactory recognition	−0.56	−0.18	.001*

Abbreviations: DG, dentate gyrus, ENT, entorhinal cortex, Para‐HI, parahippocampus, L, left, R, right.

* *p* < .05, **p < .01, ***p < .001.

## 4 DISCUSSION

A previous study showed that olfactory decline without cognitive impairment was predicted by left HI and left para‐HI volume reductions (Kubota et al., 2020). We investigated in this study the link between olfaction and volume reduction in the medial temporal regions including the para‐HI, ENT, and hippocampal subfields.

The most interesting finding in this study was that volume reduction in the ENT was not directly associated with a decline in olfactory ability. ENT volume reduction was associated with para‐HI volume reduction, and the ENT indirectly affected the olfactory ability via the para‐HI (Figure 2).

### 4.1 Medial temporal regions as a center of olfactory processing

The role of the medial temporal regions in memory function has been established (Amaral et al., 1987; Amaral & Whitter, 1989; Suzuki & Amaral, 1994; Van Hoesen & Pandya, 1975; Van Hoesen et al., 1975), and these regions act as a center of olfactory processing. Olfactory input is transmitted directly to the medial temporal regions, especially the ENT, which receives direct olfactory input (Sobel et al., 2000) and acts as a gateway to the HI for memory processing (Amaral et al., 1987). Indeed, two abilities are required for human olfaction, including detection of olfactory molecules and identification and naming of the target through memory retrieval. Damage to the ENT in AD has been reported to cause inability to detect odor and impairment of memory retrieval processing. We conjectured that volume reduction of the ENT might be related to olfactory decline, even in healthy elderly individuals. However, consistent with our previous study (Kubota et al., 2020), that the left para‐HI volume change impacted crucial on olfactory recognition rather than volume change of the ENT.

The para‐HI is connected to the hippocampal formation, particularly via the ENT. Neocortical inputs from the OFC, medial prefrontal cortex, and insular cortex are sent to the ENT, which predominately provides cortical input to the DG (Amaral et al., 1987). However, the para‐HI converges inputs from multiple modalities and projects to the ENT (Suzuki & Amaral, 1994; Van Hoesen & Pandya, 1975; Van Hoesen et al., 1975). The para‐HI also receives efferent projections from the ENT and plays a role as a hub for input and output pathways (Suzuki & Amaral, 1994). According to these anatomical and functional relationships, we suggest that impairment of olfactory recognition might result from disruption of the association between olfactory information and the context of memory retrieval, in which the para‐HI participates in convergence of information from multiple sources.

### 4.2 Relationship between the ENT and DG in olfaction

Additionally, volume reduction of the left DG was directly linked to volume reduction of the left ENT. Overall, volume reduction of the hippocampal subfields was observed in aged subjects, but the ENT volume was relatively resistant to aging. Therefore, volume reduction in the left ENT and left para‐HI was individualized and separated from hippocampal subfield volume reduction. This finding is consistent with that of Shing et al. (2011), who showed volume reduction associated with age in the CA1 region, DG, and CA3 region with relative preservation of the ENT volume.

However, among the hippocampal subfields, the left DG volume had positive correlation with the left ENT volume and indirectly affected olfactory recognition. Therefore, the degree of volume reduction of the left DG may exhibit individual differences. The ENT is directly connected to the DG, CA3 region, CA1 region, and subiculum (Amaral & Whitter, 1989). Atrophy of the DG and CA3 region is correlated with age‐related memory loss (Shing et al., 2011). The pathway between the DG and ENT has important role for associative memory information (Carr et al., 2017).

Our study only investigated the structural changes related to olfaction ability, and determination of a bi‐directional cause and effect relationship between the DG and ENT was difficult. However, reduced input from the ENT to the DG might cause decreased neurogenesis.

Newborn neurons are generated within DG and the olfactory bulb in the adult mammalian brain (Alman & Dass, 1965). In the DG, granule cells are generated throughout the life span (Altman & Das, 1965, 1967; Caviness, 1973; Gueneau et al., 1982; Kuhn et al., 1996). In addition, neurogenesis of new cells was observed in the adult human brain (Eriksson et al., 1998). Because the ENT has a direct link to the DG, the production of neurons in the DG is transiently increased by stimulation of the ENT (Stone et al., 2011). If the production of neurons is reflected as the DG volume, this volume might be affected by the functional decline of the ENT.

In addition to the left para‐HI volume reduction, a weak DG‐ENT association may lead to decreased memory processing, which might indirectly contribute to the decline in olfactory recognition in older adults.

One question may arise that olfactory decline could be caused by abnormality of the olfactory bulb. The lateral ENT may receive direct projections from the olfactory bulb (Lavenex & Amaral, 2000). In Olfactory bulb, new neurons area also generated during adulthood (Altman & Das, 1965). Possibly, less newborn neurons in the olfactory bulb may affect on the level of olfaction. However, all elderly subjects could detect odors in this study; therefore, olfactory decline may be caused by a central factor that is associated with prior volume changes of the ENT and para‐HI.

### 4.3 Laterality

The HI volume reduction associated with olfaction decline was always observed in the left hemisphere in elderly subjects (Kubota et al., 2020, and in patients with first episode of schizophrenia (Kubota et al., 2020). Another study reported that decrease of left HI volume showed an impaired visuospatial memory in patients with schizophrenia spectrum disorders (Wannan et al., et al., 2018). A meta‐analysis of structural MRI studies on the HI reported that HI asymmetry was observed in healthy control subjects, and the left hemisphere volume was lower than that of the right hemisphere (Smith, 2005).

Our results were consistent with prior findings that the left HI volume was smaller than the right HI volume in elderly and young HCs (elderly, *z *= −3.2, *p* = .001, young HCs, *z *= −4.3, *p* < .0001). This trend was not observed in the AMG in both groups; only elderly subjects had a smaller left AMG than right AMG (elderly, *z *= −3.26, *p* = .001, young HCs, *z *= −1.77, *p* = .75). It is unknown why the left HI and AMG are smaller than those of the right side, and variation in volume reduction was shown on the left side in the elderly subjects. Further study is required to investigate the functional meaning of laterality and to clarify the differences in sides.

### 4.4 Limitations

Our results were obtained from a small number of HC subjects, and additional data including patients with MCI and AD are needed to confirm our findings.

In the present study, we were unable to investigate the volume of the olfactory bulb itself because the images did not follow the protocol for olfactory bulb analysis. However, previous reports have suggested that the size of the olfactory sulcus indirectly reflects the size of the olfactory bulb (Delon‐Martin et al., 2013). We therefore compared olfactory sulcus volumes between the two groups to investigate any association between olfactory sulcus volume and olfactory ability. There were no differences in olfactory sulcus volumes between the two groups, and olfactory sulcus volume was not correlated with olfactory ability. These results indicate that the olfactory bulb may not be associated with the olfactory decline that was observed in elderly subjects in this study. It would be of interest to explore how olfactory bulb volume might relate to the central olfactory system in individuals with impaired olfaction; this concept requires further research.

In addition, the ENT was not divided into subregions in this study. The ENT has been divided into two subregions, the medial ENT and lateral ENT (Lavenex & Amaral, 2000). Khan et al. (2014) reported that the lateral ENT was affected by preclinical disease, and the dysfunction can then spread cortically. The differences in damage to the lateral ENT between AD and the preclinical condition and how this damage relates to the para‐HI and olfactory impairment will be investigated in further studies.

## CONFLICT OF INTEREST

The authors declare that this study was funded by Kao Corporation. HS was employed by Kao Corporation and was involved for MRI measurements and analysis of olfaction and MoCA data.

## AUTHOR CONTRIBUTIONS

N.I. and Y.M. designed the experiment, conducted M.R.I. experiments, and analyzed the M.R.I. data. N.I. and Y.M. wrote the initial manuscript. S.K., H.S., A.Y., and M.H. performed olfactory testing and the MoCA, and analyzed the data. N.I., H.S., S.K., M.Y., A.Y., N.K., K.W., M.I., K.O., and M.I.z. recruited participants, helped with scanning MRI, and conducted preprocessing of M.R.I data. S.K. and Sa.K. analyzed hippocampus subfield data. All authors discussed the results. Y.M. edited the final manuscript.

### Peer Review

The peer review history for this article is available at https://publons.com/publon/10.1002/brb3.2115.

## Supporting information

Supplementary Material

## Data Availability

Datasets generated for this study are available on request from the corresponding author.
