# Supplementary material for: Entorhinal cortex and parahippocampus volume reductions impact olfactory decline in aged subjects
Source: Brain Behav. 2021 Mar 26;11(5):e02115. doi: 10.1002/brb3.2115 (PMC8119819; doi:10.1002/brb3.2115)
Supplement: Supplementary file 1 — Supplementary Material [file BRB3-11-e02115-s001.doc]

**Supplemental material**

Entorhinal cortex and parahippocampus volume reductions impact olfactory decline in aged subjects

Natsuko Iizuka^1,2^, Yuri Masaoka^1*^, Satomi Kubota^2^, Haruko Sugiyama^3^, Masaki Yoshida^4^, Akira Yoshikawa^1^, Nobuyoshi Koiwa^5^, Motoyasu Honma^1^, Keiko Watanabe^2^, Shotaro Kamijo^1^, Sawa Kamimura^1^, Masahiro Ida^6^, Kenjiro Ono^2^, Masahiko Izumizaki ^1^

^1^Department of Physiology, Showa University School of Medicine, 1-5-8 Hatanodai, Shinagawaku, Tokyo, Japan, 142-8555.

^2^Division of Neurology, Department of Medicine, Showa University School of Medicine, 1-5-8 Hatanodai, Shinagawaku, Tokyo 142-8555, Japan.

^3^Sensory Science Research, Kao Corporation, 2-1-3 Bunka, Sumida-ku Tokyo 131-8501, Japan

^4^Department of Ophthalmology, Jikei Medical University, 3-25-8, Nishishinbashi, Minatoku, Tokyo 105-8461, Japan

^5^Department of Health and Science, University of Human Arts and Sciences, 1288 Magome, Iwatsukiku, Saitamashi, Saitamaken 339-8539, Japan.

^6^National Hospital Organization Mito Medical Center, 280 Sakuranosato, Ibaragicyou, higashiibaragi, Ibaragiken 311-3193, Japan

Running title: entorhinal-parahippocampus and olfaction

Correspondence:
Yuri Masaoka

[faustus@med.showa-u.ac.jp](mailto:faustus@med.showa-u.ac.jp)

Supplemental Table 1. Partial correlation between MoCA, olfactory detection, olfactory recognition and volumes of

olfactory ROI covarying with differences in age, years of education and sex in elderly subjects.

ParaHI, parahippocampus, ENT, Entorhinal cortex**,** DG, dentate gyrus, GC-DG, granule cell layer of the dentate gyrus, HATA, HI-AMG transition area, GC-ML-DG, granule cells in the molecular layer of the dentate gyrus

Supplemental Figure 1. A model created before the final path model, including the important nodes (CA1, CA3, GC-DC, and subiculum).

The CA3 and GC-DC had significant paths to the DG (P < 0.001), and the subiculum had a direct path to both the DG (P < 0.001) and the ENT (P < 0.05). However, the CA1, CA3, GC-DC, and subiculum had no indirect path to olfactory recognition, and this model had a low goodness of fit index (GFI = 0.51). The final path model was then created by eliminating the CA1, CA3, GC-DC, and subiculum (the final path model is shown in Figure 2 in the main text). DG, dentate gyrus, ENT, entorhinal cortex, para-HI, parahippocampus, GC-DC, granule cell layer of the dentate gyrus.

***P < 0.0001, **P < 0.001, *P < 0.05 (statistical details are indicated in Supplemental Table 2).

Supplemental Table 2 Statistical results of pre-final path model

Standardized direct effect

Standardized indirect effect

DG, dentate gyrus, ENT, Entorhinal cortex**,** GC-DG, granule cell layer of the dentate gyrus
